# Association between children’s intended screen time use and behavior problems in Japan: the Hokkaido Study on Environmental and Children’s Health

**DOI:** 10.1265/ehpm.25-00110

**Published:** 2025-10-24

**Authors:** Naomi Tamura, Keiko Yamazaki, Chihiro Miyashita, Atsuko Ikeda, Ammara Ajmal, Satoshi Suyama, Takashi Hikage, Manabu Omiya, Masahiro Mizuta, Reiko Kishi

**Affiliations:** 1 Center for Environmental and Health Sciences, Hokkaido University, Sapporo, Japan; 2 Faculty of Health Sciences, Hokkaido University, Sapporo, Japan; 3 Funded Research Division of Child and Adolescent Psychiatry, Hokkaido University Hospital, Sapporo, Japan; 4 Graduate School/Faculty of Information Science and Technology, Hokkaido University, Sapporo, Japan; 5 Information Initiative Center, Hokkaido University, Sapporo, Japan; 6 Center for Training Professors in Statistics, The Institute of Statistical Mathematics, Tokyo, Japan

**Keywords:** Screen behavior, Birth cohort, Children’s health, Strengths and Difficulties Questionnaire, Cross-sectional study

## Abstract

**Background:**

Long screen time hours may be associated with behavioral problems in children. To better understand the relationship between children’s behavioral problems and screen time, it the associated risk factors must be subdivided based on the purpose underlying screen use. This study examined the relationship between screen time based on intended usage and behavioral problems in Japan.

**Methods:**

This study included 3,332 children aged between 7–17 years from the Hokkaido Study on Environment and Children’s Health. From October 2020 to October 2021, the children and their parents answered questionnaires on the children’s screen use duration (never used, <30 min, ≥30 min & <1 hour, ≥1 h & <2 h, ≥2 h) based on seven intended usage categories: watching television/video, video gaming, reading books/comics, sending/receiving e-mail/messages, browsing/posting on social networking services, studying for classes/homework, drawing/editing pictures/photos/videos, along with the Strengths and Difficulties Questionnaire (SDQ). Logistic regression was used to analyze the association between screen time, purpose of children’s screen use, and behavioral problems across the 13 SDQ total scores.

**Results:**

The mean ± standard deviation age of the participants was 12.4 ± 2.4-years-old, 487 (14.6%) children were determined to have behavioral problems, and the duration of screen time increased with their age. The children’s primary purposes for screen use were watching television/video, video gaming, sending/receiving e-mail/messages, and browsing/posting on social networking services. Children who reported playing video games for ≥2 hours on weekdays had higher odds of problematic total difficulties scores than never user (Odds Ratio: 2.10, 95% confidence interval: 1.45–3.06).

**Conclusion:**

Long video gaming screen time is associated with behavioral issues, hyperactivity/inattention, and prosocial behaviors in children. Conversely, watching television and videos for 30 min–1 h per day, using e-mail or messaging, and using social networking services were significantly association with reduced odds ratio for peer relationship problems as compared to children who never engaged in these activities. Longitudinal follow-up is needed to further examine screen time and problem behaviors.

**Supplementary information:**

The online version contains supplementary material available at https://doi.org/10.1265/ehpm.25-00110.

## Graphical Abstract

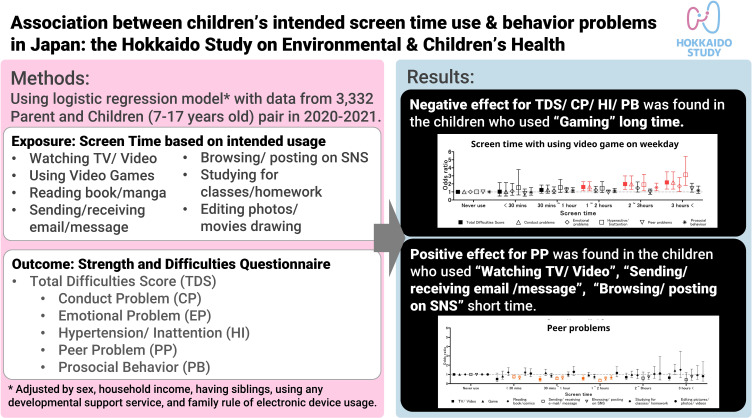

## 1. Background

Recently, the use of digital devices by children and adolescents has increased. Japan’s Ministry of Internal Affairs and Communications reported a study showing that, from 2016 to 2020, the average time teenagers spent watching television did not change: however the average time spent using the internet increased for each age group on both weekdays and weekends [1]. In addition, the coronavirus disease 2019 (COVID-19) pandemic and resultant public health actions have resulted in major changes in the daily lives of children and adolescents owing to the increased physical distance and greater time spent at home. Children and adolescents reported higher levels of electronic screen use during the pandemic than before. In a German study comparing the periods pre- and post-COVID-19, sports activity time decreased, and recreational screen time increased in children aged 4–17 years old [2].

Approximately 20% of children and adolescents experience mental health disorders, often manifested as behavioral problems [3]. These behavioral problems, even if subthreshold, can disrupt the transition to adulthood. Many pediatric mental health concerns are categorized as “behavioral problems,” which are often overlooked and inadequately addressed [4, 5]. Given their potential long-term consequences, identifying risk factors for such behaviors is crucial.

Long hours of screen time can affect children’s mental health [6, 7], as it can reduce their sleep duration, and cause them to become sedentary, reducing their physical activity [6, 8, 9]. The addictive nature of internet gaming is a growing concern in this context. The International Classification of Diseases 11th Revision (ICD-11) includes diagnostic criteria for gaming disorder [10, 11]. In many cross-sectional studies excessive screen time has been associated with behavioral problems in children [6, 12, 13]. Total daily screen time ≥2 h in children was associated with total difficulty scores [14]. However, many of these studies do not specify the screen activity performed or only count the time spent watching television as screen time. In a study on depression in children and adolescents, associations between screen behavior and depression varied according to the type of screen behavior and participant characteristics [15]. This suggests that the relationship between screen time and children’s behavioral problems differs depending on screen activity. For example, even if children look at a screen for the same amount of time, studying online classes and playing games are expected to have different associations with problematic behaviors. Previous studies have also reported changes in screen use behavior, such as children spending more time on screens on weekends than on weekdays [16].

To better understand the relationship between children’s behavioral problems and screen time, risk factors must be examined based on specific screen activities. Therefore, this study aims to clarify how different purposes of screen use influence the association between screen time and behavioral problems in children. Unlike previous studies that primarily focus on total screen duration, this study distinguishes various screen activities—including watching television/videos, video gaming, reading books/comics, messaging, browsing/posting on social networking services (SNS), studying for classes/homework, and drawing/editing pictures/photos/videos—to identify specific risks associated with each usage type. By analyzing screen use in a more nuanced manner, this study seeks to provide insights that can inform targeted interventions for reducing problematic behaviors in children.

## 2. Methods

### 2.1. Study participants

**Fig. 1:**
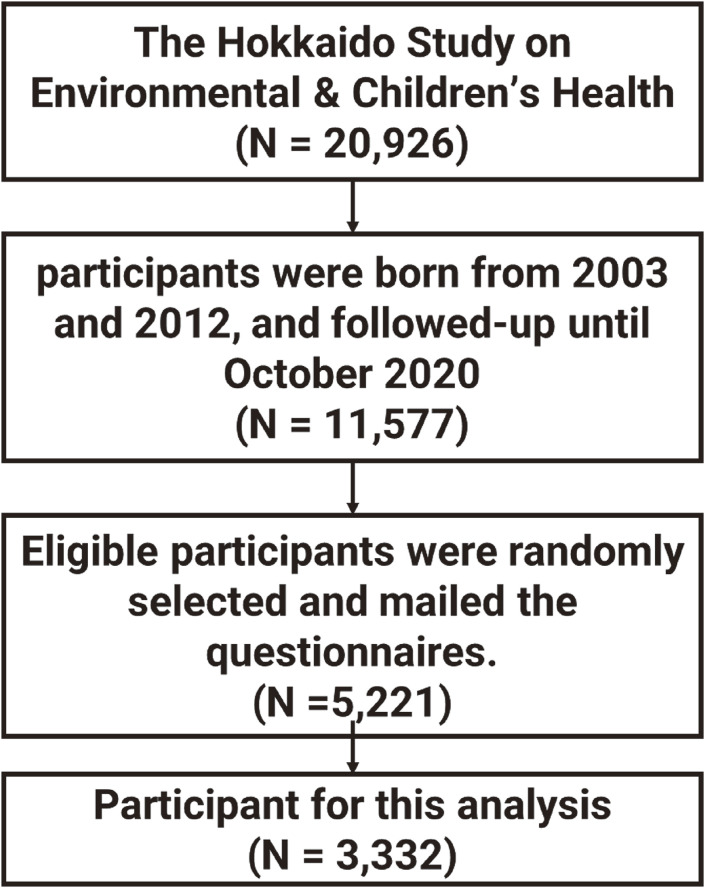
Flow chart of study participants.

In total, 3,332 children aged 7–17 years old were enrolled in this study from the Hokkaido Study on Environment and Children’s Health [17–20]. From February 2003 to March 2012, the large-scale Hokkaido cohort enrolled women during early pregnancy (13 weeks of gestational age) who visited the maternity unit in one of the cooperating hospitals and clinics in Hokkaido Prefecture for prenatal health care [17–20]. The study cohort comprised 20,926 pregnant women. From October 2020 to October 2021, using random selection, we mailed 5,221 questionnaires to parents and children. Children answered about children’s screen use duration utilizing the intended usage questionnaire, and their parents answered Japanese parent-report version of the Strengths and Difficulties Questionnaire (SDQ). Overall, 3,332 questionnaires were completed and returned (Fig. 1).

### 2.2. Outcome measurement

The SDQ is a common instrument used to assess behavioral and mental health problems among children and adolescents aged 2–17 years old [21]. The SDQ comprises 25 items, each with the option of rating not true (0), somewhat true (1), or certainly true (2). The items are divided into five subscales: conduct problems, hyperactivity/inattention, emotional problems, peer relationship problems, and prosocial behavior [21, 22]. Excluding prosocial behavior, the four subscale scores were summed up to generate the total difficulties score (TDS), which is a measure of a child/adolescent’s overall emotional and behavioral difficulties, ranging from 0 to 40. The outcome variable was TDS-based behavioral problems (<13 for normal or >=13 for borderline/high). The five subscales of the SDQ were also analyzed as outcome variables, with the following cut-off values: emotional problems: 0–3 = normal, 4 = borderline, 5–10 = clinical; conduct problems: 0–2 = normal, 3 = borderline, 4–10 = clinical; hyperactivity/inattention: 0–5 = normal, 6 = borderline, 7–10 = clinical; peer relationship problems: 0–2 = normal, 3 = borderline, 4–10 = clinical; and prosocial behavior: 6–10 = normal, 5 = borderline, 0–4 = clinical. Each subscale is dichotomized into normal (normal and borderline) and clinical groups [21].

### 2.3. Exposure measurement

The independent variables included children’s daily screen time (never used, <30 min, ≥30 min & <1 hour, ≥1 h & <2 h, and ≥2 h) on weekdays and weekends. Children reported their screen activity and duration (watching television/video, video gaming, reading books/comics, sending/receiving e-mail/messages, browsing/posting on SNS, studying for classes/homework, and drawing/editing pictures/photos/videos).

### 2.4. Covariate factors

We used the following covariates: child’s age at survey (chosen as a priority based on previous literature), sex, having siblings, the usage of any developmental support service, and having family rules for electronic devices usage. We used family household income (<3.0, 3.0–4.9, 5.0–7.9, ≥8 million yen) at pregnancy as a socioeconomic indicator. Information on these covariate factors was collected from the questionnaire.

### 2.5. Statistical analysis

Continuous data are presented as mean and standard deviation. Categorical data are presented as frequencies and percentages. Logistic regression was used to analyze the association between activity-based screen time and behavioral problems, using a crude or adjusted model with covariate factors. In the adjusted model, the number of subjects in the analysis is reduced when there are missing confounding factors. Statistical significance was set at p < 0.05. Statistical analyses were performed using R-4.3.1 for Windows (64 bit).

### 2.6. Ethical approval

Written informed consent was obtained from at least one parent of each participating child prior to enrollment in the Hokkaido Study. The study protocol was approved by the Ethics Review Board for Epidemiological Studies at Hokkaido University Graduate School of Medicine (May to March 31, 2003) and Hokkaido University Center for Environmental and Health Sciences (reference no. 19–118, August 22, 2019). This study was designed and conducted in accordance with the principles of the Declaration of Helsinki. Participants were reminded of the purpose of the study through the questionnaire distributed to them. Additionally, participants were informed that their return of the completed questionnaire was considered consent to participate in the study of electromagnetic radiation exposure and children’s health. Through these processes, participants expressed their willingness to participate in the study and were included in the analysis.

## 3. Results

The mean age of participant was 12.4 ± 2.4 years old for children, 44.1 ± 5.1 years old for mothers, and 45.8 ± 5.9 years old for fathers. Table 1 presents the characteristics of the study participants.

**Table 1:** Characteristics of study participants

		**N (%)**
Sex	Male	1663 (49.9)
	Female	1669 (50.1)
School	1^st^ to 3^rd^ grade of elementary school	366 (11.0)
	4^th^ to 6^th^ grade of elementary school	1219 (36.6)
	Junior High School	1228 (36.9)
	High school	519 (15.6)
Having siblings	No	450 (16.4)
	Yes	2298 (83.6)
Household income at birth (Million yen)	<3.0	539 (18.9)
	3.0–4.9	1314 (46.0)
	5.0–7.9	764 (26.7)
	≥8	240 (8.4)
Using any developmental support service	No	2984 (89.6)
	Yes	342 (10.3)
Having family rules for electronic devices usage	No	1454 (44.0)
	Yes	1865 (56.0)

Table 2 summarizes the distribution of children’s screen time based on intended usage as reported by the children. Over 50% of children reported the following primary screen use activities: watching television, video gaming, sending/receiving e-mail/messages, and browsing/posting on SNS. Excluding study use, the means of all screen time activities were determined to be longer on weekends than on weekdays.

**Table 2:** Distribution of children’s screen time based on intended usage, as reported by the children

**Weekday**	**Screen time (min)**	**Never use**	**<30 min**	**≥30 min & <1 hour**	**≥1 h & <2 h**	**≥2 h**
	**Mean ± SD**	**N (%)**	**N (%)**	**N (%)**	**N (%)**	**N (%)**
1. Watching TV/video	112.8 ± 85.5	126 (3.8)	58 (1.7)	368 (11.1)	1134 (34.1)	1597 (47.9)
2. Video gaming	58.2 ± 64.2	877 (26.4)	135 (4.1)	583 (17.6)	1045 (31.5)	635 (19.1)
3. Reading books/comics	14.9 ± 39.7	2363 (71.2)	197 (5.9)	336 (10.1)	243 (7.3)	108 (3.2)
4. Sending/receiving e-mail/message	16.7 ± 31.1	1673 (50.4)	719 (21.7)	514 (15.5)	279 (8.4)	93 (2.8)
5. Browsing/posting on SNS	19.1 ± 39.5	2162 (65.1)	241 (7.3)	354 (10.7)	356 (10.7)	169 (5.1)
6. Studying for classes/homework	18.3 ± 47.7	2227 (67.1)	217 (6.5)	402 (12.1)	298 (9.0)	137 (4.1)
7. Editing pictures/photos/videos	6.9 ± 24.0	2745 (82.7)	192 (5.8)	173 (5.2)	120 (3.6)	46 (1.4)

**Weekend**	**Screen time (mins)**	**Never use**	**<30 mins**	**≥30 min & <1** **hour**	**≥1 h & <2 h**	**≥2 h**
	**Mean ± SD**	**N (%)**	**N (%)**	**N (%)**	**N (%)**	**N (%)**

1. Watching TV/video	194.3 ± 141.7	90 (2.7)	20 (0.6)	125 (3.8)	542 (16.3)	2499 (75.0)
2. Video gaming	104.8 ± 117.4	673 (20.3)	89 (2.7)	348 (10.5)	818 (24.6)	1343 (40.3)
3. Reading books/comics	20.7 ± 55.9	2363 (71.2)	197 (5.9)	336 (10.1)	243 (7.3)	203 (6.1)
4. Sending/receiving e-mail/message	23.5 ± 49.2	1686 (50.8)	594 (17.9)	465 (14.0)	321 (9.7)	206 (6.2)
5. Browsing/posting on SNS	29.7 ± 63.0	2123 (63.9)	184 (5.5)	275 (8.3)	329 (9.9)	366 (11.0)
6. Studying for classes/homework	16.3 ± 40.0	2356 (70.9)	174 (5.2)	324 (9.8)	274 (8.3)	137 (4.1)
7. Editing pictures/photos/videos	11.5 ± 35.9	2635 (79.3)	167 (5.0)	188 (5.7)	163 (4.9)	114 (3.4)

SD: Standard Deviation, SNS: social networking service

Table 3 lists the results of the children’s SDQs as reported by their parents, 487 (14.6%) children were determined to have behavioral problems (i.e., borderline/high need for support), and the proportion of cases and duration of screen time increased with age.

**Table 3:** Results of children’s strength and difficulties questionnaire as reported by their parents

	**All (N = 3,332)**	**Number of cases**
	**Mean ± SD**	**N (%)**
Total Difficulties Score	7.56 ± 4.81	487 (14.6)
Conduct problems	1.59 ± 1.84	324 (9.7)
Emotional problems	1.61 ± 1.42	480 (14.4)
Hyperactivity/inattention	2.54 ± 2.01	328 (9.8)
Peer relationship problems	1.82 ± 1.64	478 (14.3)
Prosocial behavior	6.44 ± 2.13	1,111 (33.3)

SD: Standard Deviation. The outcome variable was behavioral problems according to the SDQ’s total difficulties score (<13 for normal or 13≤ for borderline-clinical cases). The five subscales of the SDQ (conduct problems, emotional problems, hyperactivity/inattention, peer relationship problems, and prosocial behavior) were analyzed as outcome variables. For the subscales, the following cut-off values were applied: emotional problems: 0–3 = normal, 4≤ borderline/clinical as cases; conduct problems: 0–3 = normal, 4≤ borderline/clinical as cases; hyperactivity/inattention: 0–5 = normal, 6≤ = borderline/clinical as cases; peer relationship problems: 0–3 = normal, 4≤ borderline/clinical as cases; and prosocial behavior: 6–10 = normal, ≤5 = borderline/clinical as cases.

Table 4-1 presents the results of the logistic regression model for associations with children’s screen time activities. In the adjusted model, for SDQ scores ≥13 the odds ratio (OR) was 2.10 (95% confidence interval [CI] 1.45–3.06) in children who played video games for ≥2 h on weekdays compared with children who never played video games. In the adjusted model there was a significant association between the OR of TDS ≥13 and playing video games, with OR increasing when game time exceeded 30 min on weekdays and 1 h on weekends, compared with that of children who never spent screen time playing video games. Table 4-2 shows the results of the logistic regression model for associations with children’s screen time activity stratified by sex. Among females the OR for children who played video games for ≥2 hours on weekdays was 2.58 (95% CI 1.46–4.50) compared to children who did not play video games, this OR was higher than that observed in males. Table 4-3 shows the results of the logistic regression model for associations with children’s screen time activity stratified by age. In junior high and high school students the OR for children who played video games for ≥2 hours on weekdays was 2.93 (95% CI 1.79–4.82) compared to those who did not play video games; this OR was higher than that observed among elementary school students.

**Table 4-1:** Associations between children’s length of screen time and behavioral problems

	**Weekday**		**Weekend**	
	**Crude**	Adjusted model*	**Crude**	Adjusted model*
	**Odds Ratio (95% CI)**	**Odds Ratio (95% CI)**	**Odds Ratio (95% CI)**	**Odds Ratio (95% CI)**
**1. Watching TV/video**				
Never use	Reference	Reference	Reference	reference
<30 min	0.88 (0.32, 2.18)	1.05 (0.36, 2.96)	1.05 (0.22, 3.69)	1.81 (0.34, 7.95)
≥30 min & <1 hour	0.67 (0.37, 1.28)	0.64 (0.30, 1.44)	0.57 (0.24, 1.34)	0.58 (0.18, 1.80)
≥1 h & <2 h	0.93 (0.55, 1.64)	0.73 (0.38, 1.51)	0.70 (0.37, 1.38)	0.74 (0.33, 1.90)
≥2 h	1.36 (0.82, 2.38)	1.12 (0.60, 2.31)	1.12 (0.64, 2.12)	1.11 (0.52, 2.72)
**2. Video gaming**				
Never use	Reference	Reference	Reference	reference
<30 min	1.01 (0.52, 1.80)	1.03 (0.46, 2.07)	0.94 (0.40, 1.92)	1.03 (0.38, 2.38)
≥30 min & <1 hour	1.31 (0.93, 1.83)	1.25 (0.84, 1.87)	1.34 (0.89, 2.02)	1.38 (0.85, 2.24)
≥1 h & <2 h	**1.78 (1.35, 2.37)**	**1.61 (1.14, 2.30)**	**1.45 (1.05, 2.02)**	1.39 (0.93, 2.09)
≥2 h	**2.79 (2.09, 3.75)**	**2.10 (1.45, 3.06)**	**2.24 (1.69, 3.02)**	**1.81 (1.25, 2.65)**
**3. Reading books/comics**				
Never use	Reference	Reference	Reference	reference
<30 min	1.24 (0.83, 1.82)	1.45 (0.89, 2.27)	1.47 (0.94, 2.20)	**1.83 (1.09, 2.98)**
≥30 min & <1 hour	1.16 (0.84, 1.58)	1.25 (0.85, 1.81)	**0.62 (0.40, 0.91)**	0.75 (0.46, 1.18)
≥1 h & <2 h	1.12 (0.78, 1.58)	1.19 (0.77, 1.79)	**1.43 (1.04, 1.95)**	**1.47 (1.00, 2.12)**
≥2 h	1.49 (0.89, 2.39)	1.46 (0.75, 2.67)	1.17 (0.78, 1.70)	1.26 (0.75, 2.04)
**4. Sending/receiving e-mail/message**				
Never use	Reference	Reference	Reference	reference
<30 min	**0.75 (0.58, 0.96)**	0.95 (0.68, 1.31)	0.81 (0.61, 1.05)	1.10 (0.78, 1.53)
≥30 min & <1 hour	**0.74 (0.55, 0.98)**	1.07 (0.72, 1.55)	**0.71 (0.52, 0.96)**	0.98 (0.66, 1.45)
≥1 h & <2 h	0.82 (0.56, 1.16)	1.21 (0.74, 1.94)	0.80 (0.56, 1.12)	1.15 (0.72, 1.79)
≥2 h	1.07 (0.60, 1.82)	1.36 (0.63, 2.70)	0.78 (0.50, 1.17)	0.95 (0.52, 1.64)
**5. Browsing/posting on SNS**				
Never use	Reference	Reference	Reference	reference
<30 min	0.69 (0.45, 1.03)	0.80 (0.47, 1.29)	0.94 (0.61, 1.41)	1.00 (0.57, 1.69)
≥30 min & <1 hour	**0.62 (0.42, 0.87)**	0.97 (0.62, 1.47)	**0.59 (0.39, 0.88)**	0.88 (0.54, 1.38)
≥1 h & <2 h	0.75 (0.53, 1.03)	1.09 (0.70, 1.67)	**0.64 (0.44, 0.92)**	0.89 (0.56, 1.39)
≥2 h	1.00 (0.64, 1.52)	1.19 (0.66, 2.06)	0.77 (0.55, 1.06)	1.01 (0.65, 1.54)
**6. Studying for classes/homework**				
Never use	Reference	Reference	Reference	reference
<30 min	1.02 (0.68, 1.48)	1.02 (0.63, 1.60)	0.96 (0.60, 1.46)	0.85 (0.49, 1.40)
≥30 min & <1 hour	1.02 (0.75, 1.36)	1.23 (0.86, 1.73)	1.04 (0.75, 1.43)	1.23 (0.83, 1.79)
≥1 h & <2 h	0.76 (0.51, 1.08)	0.82 (0.51, 1.27)	0.84 (0.57, 1.20)	0.82 (0.49, 1.30)
≥2 h	0.86 (0.50, 1.40)	0.74 (0.35, 1.42)	0.70 (0.39, 1.18)	0.89 (0.45, 1.63)
**7. Editing pictures/photos/videos**				
Never use	Reference	Reference	Reference	reference
<30 min	1.05 (0.68, 1.56)	1.11 (0.65, 1.80)	0.92 (0.57, 1.43)	1.11 (0.63, 1.87)
≥30 min & <1 hour	1.29 (0.84, 1.91)	1.08 (0.62, 1.79)	1.39 (0.93, 2.01)	0.94 (0.52, 1.59)
≥1 h & <2 h	**1.95 (1.25, 2.97)**	**2.34 (1.36, 3.89)**	1.26 (0.81, 1.89)	1.52 (0.92, 2.44)
≥2 h	0.92 (0.35, 2.02)	1.39 (0.51, 3.23)	1.29 (0.77, 2.07)	1.40 (0.73, 2.49)

*Adjusted for child’s age at survey, sex, household income, having siblings, and using any developmental support service, and family rule of electronic device usage.

**Table 4-2:** Associations between children’s length of screen time and behavioral problems stratified by children’s sex

	**Male**		**Female**	
	**Crude**	Adjusted model*	**Crude**	Adjusted model*
	**Odds Ratio (95% CI)**	**Odds Ratio (95% CI)**	**Odds Ratio (95% CI)**	**Odds Ratio (95% CI)**
**1. Watching TV/video**				
Never use	reference	Reference	Reference	reference
<30 min	0.82 (0.17, 3.12)	1.34 (0.24, 6.49)	1.08 (0.27, 3.82)	0.95 (0.21, 4.00)
≥30 min & <1 hour	0.62 (0.28, 1.47)	0.76 (0.28, 2.32)	0.64 (0.25, 1.78)	0.44 (0.13, 1.59)
≥1 h & <2 h	0.74 (0.37, 1.62)	0.64 (0.26, 1.81)	1.10 (0.52, 2.73)	0.83 (0.33, 2.55)
≥2 h	0.95 (0.48, 2.04)	0.93 (0.39, 2.59)	1.84 (0.88, 4.48)	1.43 (0.58, 4.30)
**2. Video gaming**				
Never use	reference	Reference	Reference	reference
<30 min	1.67 (0.63, 3.97)	1.28 (0.28, 4.16)	0.69 (0.26, 1.53)	1.12 (0.41, 2.61)
≥30 min & <1 hour	1.12 (0.65, 1.95)	1.20 (0.64, 2.28)	1.37 (0.89, 2.10)	1.38 (0.81, 2.33)
≥1 h & <2 h	1.50 (0.97, 2.38)	1.40 (0.84, 2.42)	**1.83 (1.24, 2.71)**	**2.03 (1.25, 3.30)**
≥2 h	**2.29 (1.48, 3.65)**	**1.88 (1.11, 3.29)**	**2.96 (1.88, 4.64)**	**2.58 (1.46, 4.50)**
**3. Reading books/comics**				
Never use	reference	Reference	Reference	reference
<30 min	1.34 (0.79, 2.18)	**2.04 (1.07, 3.71)**	1.12 (0.57, 2.03)	1.07 (0.48, 2.16)
≥30 min & <1 hour	1.01 (0.65, 1.54)	1.26 (0.75, 2.05)	1.37 (0.84, 2.15)	1.28 (0.70, 2.21)
≥1 h & <2 h	1.02 (0.62, 1.62)	1.15 (0.62, 2.04)	1.28 (0.74, 2.10)	1.27 (0.67, 2.27)
≥2 h	0.45 (0.15, 1.02)	0.61 (0.17, 1.64)	**4.07 (2.13, 7.52)**	**3.12 (1.30, 6.99)**
**4. Sending/receiving e-mail/message**				
Never use	reference	Reference	Reference	reference
<30 min	**0.67 (0.46, 0.95)**	0.93 (0.58, 1.46)	0.96 (0.66, 1.39)	0.98 (0.60, 1.57)
≥30 min & <1 hour	0.73 (0.49, 1.07)	1.16 (0.68, 1.92)	0.82 (0.51, 1.27)	1.02 (0.57, 1.78)
≥1 h & <2 h	0.62 (0.34, 1.07)	1.09 (0.50, 2.21)	1.17 (0.70, 1.89)	1.29 (0.66, 2.44)
≥2 h	0.75 (0.25, 1.81)	1.06 (0.23, 3.48)	1.60 (0.77, 3.09)	1.50 (0.59, 3.50)
**5. Browsing/posting on SNS**				
Never use	reference	Reference	Reference	reference
<30 min	0.96 (0.56, 1.57)	1.39 (0.72, 2.56)	**0.49 (0.23, 0.94)**	**0.34 (0.12, 0.81)**
≥30 min & <1 hour	0.64 (0.36, 1.06)	1.09 (0.55, 2.05)	0.68 (0.40, 1.10)	0.86 (0.47, 1.51)
≥1 h & <2 h	**0.50 (0.27, 0.87)**	0.73 (0.32, 1.50)	1.07 (0.69, 1.62)	1.25 (0.71, 2.14)
≥2 h	0.85 (0.34, 1.81)	0.81 (0.18, 2.48)	1.29 (0.75, 2.12)	1.20 (0.60, 2.26)
**6. Studying for classes/homework**				
Never use	reference	Reference	Reference	reference
<30 min	1.26 (0.74, 2.04)	1.43 (0.79, 2.51)	0.81 (0.41, 1.45)	0.56 (0.22, 1.20)
≥30 min & <1 hour	0.96 (0.63, 1.42)	1.03 (0.63, 1.65)	1.11 (0.70, 1.69)	1.54 (0.91, 2.52)
≥1 h & <2 h	0.84 (0.50, 1.35)	0.94 (0.48, 1.70)	0.70 (0.39, 1.20)	0.69 (0.33, 1.32)
≥2 h	0.66 (0.29, 1.33)	0.47 (0.13, 1.26)	1.15 (0.54, 2.20)	0.99 (0.36, 2.27)
**7. Editing pictures/photos/videos**				
Never use	reference	Reference	Reference	reference
<30 min	1.65 (0.86, 2.99)	1.45 (0.66, 2.98)	0.96 (0.51, 1.65)	1.05 (0.49, 2.06)
≥30 min & <1 hour	0.90 (0.39, 1.83)	0.58 (0.17, 1.55)	**1.85 (1.10, 2.98)**	1.45 (0.76, 2.62)
≥1 h & <2 h	0.68 (0.20, 1.75)	0.39 (0.06, 1.49)	**3.36 (2.01, 5.46)**	**3.64 (2.01, 6.40)**
≥2 h	1.65 (0.36, 5.57)	2.20 (0.44, 8.75)	0.81 (0.19, 2.29)	1.01 (0.23, 3.03)

*Adjusted for child’s age at survey, household income, having siblings, and using any developmental support service, and family rule of electronic device usage.

**Table 4-3:** Associations between children’s length of screen time and behavioral problems stratified by children’s age

	**Elementary School Students**		**Junior High and High School Students**	
	**Crude**	Adjusted model*	**Crude**	Adjusted model*
	**Odds Ratio (95% CI)**	**Odds Ratio (95% CI)**	**Odds Ratio (95% CI)**	**Odds Ratio (95% CI)**
**1. Watching TV/video**				
Never use	Reference	Reference	Reference	Reference
<30 min	0.45 (0.07, 1.93)	0.79 (0.10, 4.38)	1.39 (0.39, 4.56)	1.27 (0.33, 4.75)
≥30 min & <1 hour	0.68 (0.29, 1.70)	0.96 (0.31, 3.41)	0.70 (0.30, 1.76)	0.50 (0.18, 1.49)
≥1 h & <2 h	0.87 (0.43, 1.96)	0.84 (0.32, 2.69)	0.97 (0.47, 2.28)	0.70 (0.30, 1.93)
≥2 h	1.30 (0.65, 2.87)	1.32 (0.51, 4.13)	1.41 (0.70, 3.27)	1.05 (0.46, 2.86)
**2. Video gaming**				
Never use	Reference	Reference	Reference	Reference
<30 min	0.90 (0.35, 2.00)	0.70 (0.16, 2.19)	1.03 (0.38, 2.34)	1.46 (0.53, 3.43)
≥30 min & <1 hour	0.96 (0.59, 1.57)	0.92 (0.49, 1.70)	**1.64 (1.03, 2.60)**	1.69 (0.99, 2.86)
≥1 h & <2 h	**1.62 (1.09, 2.44)**	1.48 (0.88, 2.54)	**1.71 (1.15, 2.56)**	**1.75 (1.08, 2.84)**
≥2 h	**2.10 (1.37, 3.26)**	1.46 (0.82, 2.62)	**3.41 (2.31, 5.09)**	**2.93 (1.79, 4.82)**
**3. Reading books/comics**				
Never use	Reference	Reference	Reference	Reference
<30 min	1.54 (0.87, 2.59)	1.90 (0.95, 3.62)	1.05 (0.57, 1.80)	1.11 (0.54, 2.10)
≥30 min & <1 hour	1.31 (0.79, 2.08)	1.47 (0.80, 2.59)	1.12 (0.72, 1.68)	1.10 (0.66, 1.77)
≥1 h & <2 h	1.19 (0.69, 1.96)	1.11 (0.53, 2.13)	1.12 (0.68, 1.76)	1.20 (0.68, 2.01)
≥2 h	1.54 (0.68, 3.13)	1.99 (0.71, 4.95)	1.52 (0.76, 2.82)	1.17 (0.46, 2.61)
**4. Sending/receiving e-mail/message**				
Never use	Reference	Reference	Reference	Reference
<30 min	0.91 (0.61, 1.33)	1.15 (0.69, 1.86)	0.71 (0.49, 1.03)	0.81 (0.52, 1.26)
≥30 min & <1 hour	0.76 (0.39, 1.36)	1.10 (0.52, 2.15)	0.77 (0.53, 1.13)	0.95 (0.60, 1.49)
≥1 h & <2 h	1.19 (0.34, 3.25)	1.08 (0.16, 4.12)	0.84 (0.53, 1.29)	1.06 (0.62, 1.76)
≥2 h	1.01 (0.05, 6.31)	0.56 (0.03, 4.44)	1.15 (0.60, 2.07)	1.27 (0.56, 2.62)
**5. Browsing/posting on SNS**				
Never use	Reference	Reference	Reference	Reference
<30 min	0.73 (0.30, 1.53)	0.56 (0.16, 1.52)	0.77 (0.46, 1.25)	0.90 (0.50, 1.57)
≥30 min & <1 hour	0.53 (0.22, 1.08)	0.84 (0.31, 1.92)	0.73 (0.47, 1.10)	1.01 (0.61, 1.63)
≥1 h & <2 h	0.31 (0.08, 0.86)	0.42 (0.07, 1.43)	0.95 (0.64, 1.38)	1.22 (0.76, 1.94)
≥2 h	0.77 (0.18, 2.28)	0.37 (0.02, 1.93)	1.19 (0.72, 1.90)	1.32 (0.71, 2.36)
**6. Studying for classes/homework**				
Never use	Reference	Reference	Reference	Reference
<30 min	1.14 (0.68, 1.85)	1.14 (0.58, 2.12)	0.85 (0.43, 1.52)	0.91 (0.44, 1.73)
≥30 min & <1 hour	1.13 (0.75, 1.65)	1.37 (0.84, 2.20)	0.88 (0.54, 1.36)	1.11 (0.65, 1.83)
≥1 h & <2 h	0.72 (0.39, 1.26)	0.63 (0.27, 1.30)	0.80 (0.48, 1.26)	0.94 (0.51, 1.61)
≥2 h	1.07 (0.31, 2.86)	1.74 (0.50, 5.30)	0.59 (0.29, 1.11)	0.46 (0.16, 1.08)
**7. Editing pictures/photos/videos**				
Never use	Reference	Reference	Reference	Reference
<30 min	1.02 (0.56, 1.72)	1.36 (0.65, 2.70)	1.05 (0.54, 1.90)	0.90 (0.38, 1.85)
≥30 min & <1 hour	0.93 (0.50, 1.63)	0.64 (0.24, 1.46)	**1.78 (0.98, 3.08)**	1.71 (0.85, 3.22)
≥1 h & <2 h	1.69 (0.86, 3.13)	**3.01 (1.31, 6.44)**	**2.25 (1.22, 3.95)**	**2.10 (1.02, 4.07)**
≥2 h	1.45 (0.33, 4.71)	2.72 (0.54, 10.32)	0.73 (0.17, 2.07)	1.05 (0.24, 3.14)

*Adjusted for sex, household income, having siblings, and using any developmental support service, and family rule of electronic device usage.

The results of subscale analysis indicated that in the adjusted model was shown in Fig. 2-1, 2-2 and Supplemental Table 2 to 7. The OR of conduct problems was 1.59 (95%CI: 1.02–2.49) and for hyperactivity/inattention, it was 2.41 (95%CI: 1.52–3.88) for children who used video games for ≥2 h on weekdays compared with those who never spent screen time playing video games. In the adjusted model, the OR for having an SDQ score indicating prosocial behavior was 1.40 (95% CI: 1.06–1.83) for children who played video games for ≥2 h on weekdays compared with those who never spent screen time playing video games. The ORs for the SDQ score of conduct problems, and hyperactivity/inattention tended to increase with increasing screen time. In all cases, the ORs for weekdays were higher than those for weekends.

**Fig. 2-1:**
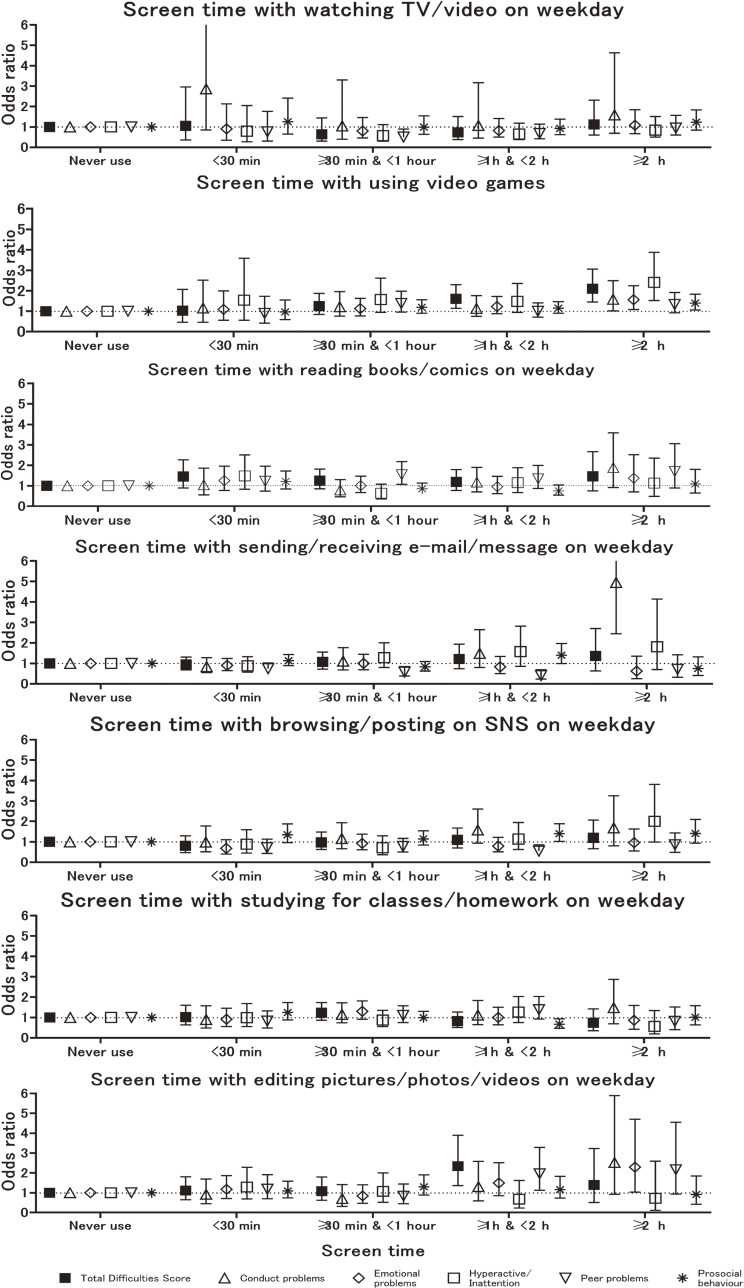
Logistic regression results for associations between children’s screen time and SDQ on weekdays. Note: mins: minutes, TV: television, SDQ: Strength and Difficulties Questionnaire, SNS: Social Networking Service. The bars show 95% confidence intervals. Peer relationship problems. Odds ratios were calculated for borderline and clinical cases in the SDQ using non-screen users as a reference. Symbols represent odds ratios for different SDQ scales: ■ (total difficulties), △ (conduct problems), ◇ (hyperactivity/inattention), □ (emotional problems), ▽ (peer relationship problems), and * (prosocial behavior).

**Fig. 2-2:**
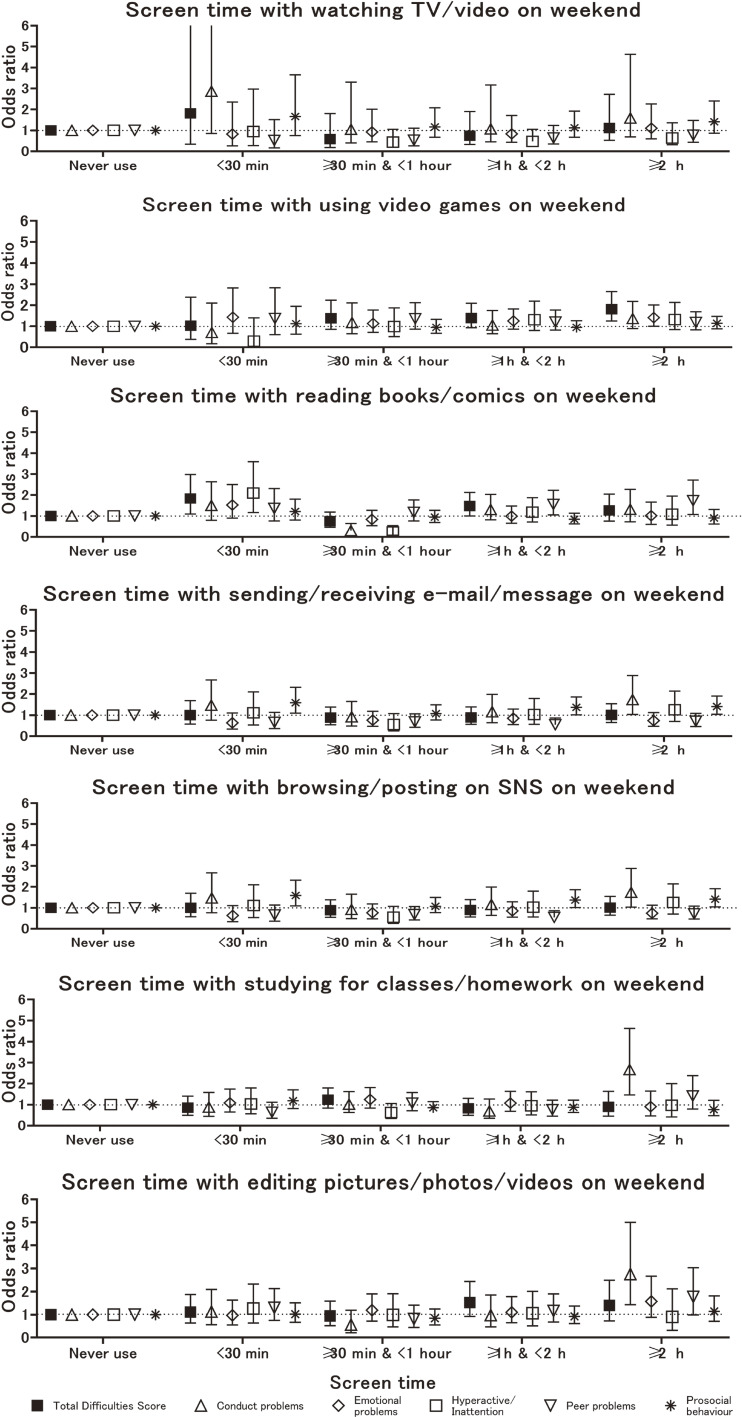
Logistic regression results for associations between children’s screen time and SDQ on weekends. Note: mins: minutes, TV: television, SDQ: Strength and Difficulties Questionnaire, SNS: Social Networking Service. The bars show 95% confidence intervals. Odds ratios were calculated for borderline and clinical cases in the SDQ using non-screen users as a reference. Symbols represent odds ratios for different SDQ scales: ■ (total difficulties), △ (conduct problems), ◇ (hyperactivity/inattention), □ (emotional problems), ▽ (peer relationship problems), and * (prosocial behavior).

Additionally, subscale analysis in the adjusted model, the OR of peer relationship problems was 0.40 (95% CI: 0.23–0.69) for children who used e-mails/messages for ≥1 hour & <2 hours on weekdays compared with those children who never spent screen time sending e-mail/messages (Fig. 2-1 and Supplemental Table 4). In the adjusted model, the OR of having an SDQ score indicating peer relationship problems was significantly lower 0.54 (95% CI: 0.33–0.85) for children who used SNS for ≥1 hour & <2 hours on weekdays compared with those who did not (Fig. 2-1 and Supplemental Table 5).

## 4. Discussion

### 4.1. Summary

This study examined the relationship between the duration of screen time and behavioral problems in children, based on the intended purpose of screen use. The result of this present study is comparable to the distribution reported in a previous study by Moriwaki et al. [23], suggesting that our findings can be generalized across the broad population of Japanese children [23].

### 4.2. Screen time with TDS and subscale scores

In the current study, excessive screen time spent playing video games was significantly associated with a higher OR of children having an SDQ score indicative of behavioral problems. The World Health Organization guidelines on physical activity, sedentary behavior, and sleep for children aged <5 years recommend that their screen time should not exceed 1 h per day, and that less is better [24, 25]. Canadian guidelines for school-age children recommend <2 h of screen time per day [26]. Compared with children who did not engage in excessive screen time, in the present study there was an increase in the ORs of TDS significantly associated with children playing video games for >1 h on weekdays and >2 h on weekends. However, the most common type of screen use in children was watching television and videos, which was not significantly associated with behavioral problems, even after ≥2 hours of screen time.

Although the numerous previous studies have evaluated screen time for across various digital devices [6, 12, 14, 27], it remains difficult to compare our results with them. Research on internet addiction has shown that, while no significant differences were detected in autism spectrum and intelligence quotients between internet addiction and control groups, the SDQ scores indicated higher attention deficit hyperactivity disorder symptom rates in the internet addiction group than in the control group [28]. In a study on depression in children and adolescents, greater time spent engaging in newer types of screen behaviors, including social media, online games, and online videos was associated with a higher prevalence of depression, whereas more time spent watching television was associated with a lower prevalence of depression [15]. These findings suggest that the relationship between screen time and children’s behavioral problems differs depending on screen activity. Therefore, electronic devices with screens may be essential communication tools for children, similar to those used by adults.

### 4.3. Effects of children’s sex and school group on behavioral problems and screen time

Moriwaki et al. [23] reported that boys scored significantly higher than girls in TDS, conduct problems, hyperactivity and inattention, and peer problems subscales. They also found that the TDS in the SDQ tended to be higher in younger children [23]. These findings suggest that sex and age may be important confounding factors when investigating problem behaviors. Therefore, a stratified analysis was conducted in the current study. The results indicated that the effects of screen time on behavioral problems varied depending on the child’s gender and age. Kawabe et al. [28] reported that males engaged in gaming more frequently than females. Interestingly, despite females being considered less frequent gamers, the relationship between children’s screen time and SDQ revealed that the OR for prolonged gaming was higher in females.

### 4.4. Weekday vs weekend

In this study, the means of all screen times were longer on weekends than on weekdays, except for use for study purposes. However, the ORs for weekdays were higher than those for weekends in all cases. For instance, compared with children who never used screen time to play video games, the ORs of TDS were 2.10 (95% CI: 1.45–3.06) for children who played video games for ≥2 h on weekdays, and 1.81 (95% CI: 1.25–2.65) on weekends. Previous studies have reported that children spend more time on screens during weekends than on weekdays [29], which is consistent with our results. The use of screens to play video games for longer periods on weekdays, when time is limited due to the school commute, may have a serious impact on problematic behavior. Therefore, it is necessary to consider ways to prevent screen time spent on video games from affecting children’s health, such as by adding different time limits for use on weekdays and weekends.

### 4.5. Strengths and limitations

This study included had large sample size as it involved children aged 7–17 years old, covering various school ages. Screen time was evaluated in detail based on activity and was further divided into weekdays and weekends. This study was conducted between 2020 and 2021, enabling the examination of recent data on the relationship between children’s mobile device use and behavioral problems. In this study, screen time was based on children’s self-reports, which may have been under- or over-reported compared to data provided by objective measurements. The results accounted for interrelated variables and potential confounding factors, including family financial status, family composition, and use of developmental support services. However, caution is needed in interpreting the results, as other important confounding factors and contextual factors such as parenting style, parental screen habits, sleep quality and COVID-19-related stress factors were not considered. Notably, data collection was conducted between 2020 and 2021 and may have been influenced by the COVID-19 pandemic. The evaluation of screen time usage in this study did not account for the possibility that children may use multiple electronic devices simultaneously. This limitation highlights the need for further consideration in future research. Despite these limitations, this study provides a candidate purpose for the use of screen time to influence problem behavior. Future Longitudinal follow-up is needed to further examine screen time and problem behavior, and the potential contribution of electromagnetic radiation to problem behavior.

## 5. Conclusion

This study examined the relationship between the length of screen time and behavioral problems based on intended use. A significant increase in OR was associated with prolonged video game screen use. Additionally, the OR of the SDQ scores indicated conduct problems, hyperactivity/inattention, and prosocial behaviors. In contrast, compared to children who did not, engage in these activities, those who used e-mails or sent messages on SNS had significantly reduced ORs for peer relationship problems. Longitudinal follow-up studies are needed for in-depth comprehension regarding the association between screen time and problem behaviors.

## Abbreviations

CI: Confidence Interval
GIGA: Global and Innovation Gateway for All
ICD-11: The 11th edition of the International Classification of Diseases
OR: Odds Ratio
SD: Standard Deviation
SDQ: Strengths and Difficulties Questionnaire
SNS: Social Networking Service
TDS: Total Difficulties Score

## Supplementary information

**Additional file 1:** **Supplemental Table 1:** Associations between children’s screen time for watching TV/video and behavioral problems subscale. **Supplemental Table 2:** Associations between children’s video gaming screen time and behavioral problems. **Supplemental Table 3:** Associations between children’s screen time for reading books/comics and behavioral problems subscale. **Supplemental Table 4:** Associations between children’s screen time for sending/receiving e-mail messages and behavioral problems subscale. **Supplemental Table 5:** Associations between children’s screen time for browsing/posting on SNS and behavioral problem subscales. **Supplemental Table 6:** Associations between children’s screen time for studying classes/homework and behavioral problem subscales. **Supplemental Table 7:** Associations between children’s screen time for editing pictures/photos/videos and behavioral problem subscales.

## Declarations

### Availability of data and material

The datasets generated and/or analyzed during the current study are not publicly available because the study involves human participants with a non-disclosure provision of individual data stated in the written informed consent to prevent compromise of study participants’ privacy but are available from the corresponding author upon reasonable request.
