# Supplementary material for: Association between children’s intended screen time use and behavior problems in Japan: the Hokkaido Study on Environmental and Children’s Health
Source: Environ Health Prev Med. 2025 Oct 24;30:82. doi: 10.1265/ehpm.25-00110 (PMC12583968; doi:10.1265/ehpm.25-00110)
Supplement: Supplementary file 1 — Additional file 1: Supplemental Table 1: Associations between children’s screen time for watching TV/video and behavioral problems subscale. Supplemental Table 2: Associations between children’s video gaming screen time and behavioral problems. Supplemental Table 3: Associations between children’s screen time for reading books/comics and behavioral problems subscale. Supplemental Table 4: Associations between children’s screen time for sending/receiving e-mail messages and behavioral problems subscale. Supplemental Table 5: Associations between children’s screen time for browsing/posting on SNS and behavioral problem subscales. Supplemental Table 6: Associations between children’s screen time for studying classes/homework and behavioral problem subscales. Supplemental Table 7: Associations between children’s screen time for editing pictures/photos/videos and behavioral problem subscales. [file ehpm-30-082-s001.docx]

Supplemental Table 1: Associations between children’s screen time for watching TV/video and behavioral problems subscale

* Adjusted for child's age at time of survey, sex, household income, having siblings, using any developmental support service, and family rule of electronic device usage.

Supplemental Table 2: Associations between children’s video gaming screen time and behavioral problems

* Adjusted for child's age at survey, sex, household income, having siblings, using any developmental support service, and family rule of electronic device usage.

Supplemental Table 3: Associations between children’s screen time for reading books/comics and behavioral problems subscale

* Adjusted for child's age at survey, sex, household income, having siblings, using any developmental support service, and family rule of electronic device usage.

Supplemental Table 4: Associations between children’s screen time for sending/receiving e-mail messages and behavioral problems subscale

* Adjusted for child's age at survey, sex, household income, having siblings, using any developmental support service, and family rule of electronic device usage.

Supplemental Table 5: Associations between children’s screen time for browsing/posting on SNS and behavioral problem subscales

* Adjusted for child's age survey, sex, household income, having siblings, using any developmental support service, and family rule of electronic device usage. SNS: social networking service.

Supplemental Table 6: Associations between children’s screen time for studying classes/homework and behavioral problem subscales

* Adjusted for child's age at survey, sex, household income, having siblings, using any developmental support service, and family rule of electronic device usage.

Supplemental Table 7: Associations between children’s screen time for editing pictures/photos/videos and behavioral problem subscales

* Adjusted for child's age at survey, sex, household income, having siblings, using any developmental support service, and family rule of electronic device usage.
